# Monitoring the COVID-19 Vaccine Acceptance Trend and its Determinants Among Iranian Adults

**DOI:** 10.34172/aim.2023.65

**Published:** 2023-08-01

**Authors:** Leila Molaeipour, Ahmad Hajebi, Leila Janani, Masoud Salehi, Mohammad Hossein Taghdisi, Hajar Nazari-Kangavari, Neda Esmailzadehha, Fatemeh Varse, Nasrin Pourattar, Seyed Abbas Motevalian

**Affiliations:** ^1^Department of Epidemiology, School of Public Health, Iran University of Medical Sciences, Tehran, Iran; ^2^Research Center for Addiction and Risky Behaviors (ReCARB), Psychosocial Health Research Institute (PHRI), Iran University of Medical Sciences, Tehran, Iran; ^3^Department of Psychiatry, School of Medicine, Iran University of Medical Sciences, Tehran, Iran; ^4^Imperial Clinical Trials Unit, School of Public Health, Faculty of Medicine, Imperial College London, London, United Kingdom; ^5^Department of Biostatistics, School of Public Health, Iran University of Medical Sciences, Tehran, Iran; ^6^Public Health Department, Faculty of Health and Medical Engineering, Tehran Medical Sciences, Islamic Azad University, Tehran, Iran

**Keywords:** COVID-19, COVID-19 vaccines, Iran, SARS-CoV-2, Vaccines

## Abstract

**Background:**

Vaccination seems to be the most critical means of halting the COVID-19 pandemic. It is crucial to understand the factors that influence COVID-19 vaccine acceptance to avoid low vaccination rates. This study intended to monitor the COVID-19 vaccine acceptance and its association with socio-demographic factors and prior diagnosis of COVID-19 in Iranian adults during the COVID-19 pandemic.

**Methods:**

The study utilized data from the COVID-19 Population Survey of Iran (COPSIR), a repeated national survey designed to monitor COVID-19-related behavioral insights. From April 2020 to November 2021, thirteen iterations of a series of cross-sectional studies involving computer-assisted telephone interviews were conducted.

**Results:**

The COVID-19 vaccine acceptance rate remained above 80% until the ninth wave in February 2021, when it dropped to 62.9%. However, throughout the next four surveys, it rose gradually from 72.0% to 85.7%. The multilevel regression model revealed that the COVID-19 vaccine acceptance was significantly and positively linked with age and education.

**Conclusion:**

Despite the relatively high COVID-19 vaccine acceptance rate among Iranian adults, after the emergence of vaccines on the global market and controversies about their safety in Iran, the initially high vaccine acceptance rate dropped significantly, and then increased over time and returned to its peak level (85%). According to the Commodity Theory, this rise in vaccine acceptance can be attributed to the COVID-19 vaccine shortage in the country between January and July 2022. For Iranian adults to accept vaccines more readily, health promotion programs should target the youth and the less literate adults.

## Introduction

 The COVID-19 pandemic gravely jeopardized global health and severely disrupted the global economy, and vaccination is the best hope for stopping the pandemic. Governments are attempting to provide their populations with approved vaccines. From the start of the COVID-19 vaccination in December 2020 to the end of April 2022, 65.4% of the world’s population have received at least one dose of a COVID-19 vaccine,^1^ whereas only 15.7% of the population in low-income nations have received at least one dose.^2^

 After months of vaccination, vaccine coverage in most nations has not yet reached the level required for herd immunity, and COVID-19 vaccine hesitancy has become a worldwide health concern. Numerous studies have demonstrated that vaccination hesitancy varies by location and time.^3^ A systematic review published in 2022 revealed that South America had the highest vaccine acceptance rate (78.4%) among world regions, while Africa had the lowest (56.6%). Moreover, a cross-national comparison of vaccine acceptance rates revealed the highest rates among Ecuadorian adults (97.0%), Malaysian adults (94.3%), and Indonesian adults (93.3%), whereas the lowest percentage was recorded among Lebanese adults (21.0%).^4^ Some studies have reported a decline in vaccine acceptance during the COVID-19 pandemic.^5^ Likewise, a rapid systematic review revealed that vaccine acceptance dropped from 70% in March 2020 to below 50% in October 2020.^6^

 Numerous studies have been conducted across the globe to determine the socio-demographic factors that influence the COVID-19 vaccine acceptance; however, their findings have been inconsistent. For instance, some researchers have found that socio-demographic factors such as age, gender, education, marital status, occupation, and ethnicity are linked with vaccine acceptance.^5,7,8^ Contrarily, other studies have failed to support these associations.^9,10^

**Figure 1:**
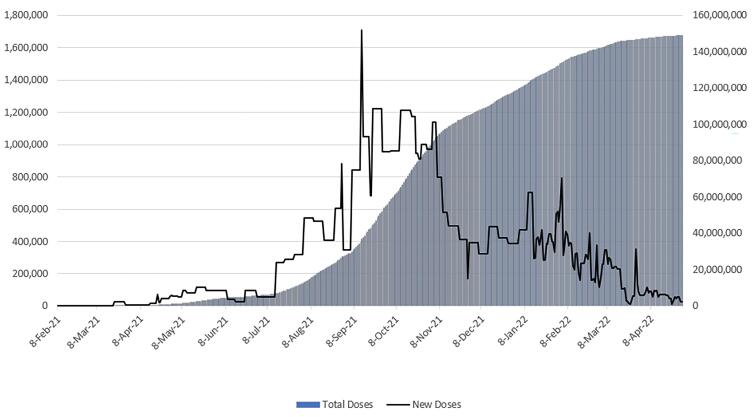
COVID-19 Vaccine Daily New Doses (Left) and Total Doses (Right) in Iran, February 2021–April 2022

 The first dose of the COVID-19 vaccine was inoculated in Iran on February 17, 2021. By the end of April 2022, nearly 149 million doses of the COVID-19 vaccine had been administered (Figure 1). Seventy-six percent of Iranians have received at least one dose of the COVID-19 vaccine, and 69% are fully vaccinated.^1^ From February to mid-July 2021, as illustrated in Figure 1, the COVID-19 vaccination rollout progressed slowly because of vaccine shortage. Nonetheless, vaccine coverage is contingent on vaccine acceptance, even if sufficient vaccines are available. As a result, assessing COVID-19 vaccine acceptance during the pandemic is critical in explaining the trend of community vaccination rollout. It is essential to comprehend the COVID-19 vaccine acceptance rate and identify the influencing factors. This information can help health decision-makers adopt strategies that minimize vaccine hesitancy. Accordingly, this study was undertaken to assess the COVID-19 vaccine acceptance rate and its determinants among Iranian adults from April 2020 to November 2021.

## Materials and Methods

###  Study Design

 This study utilized data from the COVID-19 Population Survey of Iran (COPSIR), which is a localized version of the COVID-19 Snapshot MOnitoring (COSMO) study.^11^ This serial cross-sectional study was conducted in 13 waves from April 2020 to November 2021. A survey of Iranian adults aged 18 years and older was conducted during the COVID-19 pandemic to monitor the trends of COVID-19 knowledge, preventive behaviors (including personal health behaviors, physical distancing behaviors, and COVID-19 vaccine acceptance), risk perception, public trust, and psychological problems during the COVID-19 pandemic. The 1st to 13th waves of COPSIR have been approved by the ethics committee of Iran university of medical sciences (IR.IUMS.REC.1399.004 and IR.IUMS.REC.1399.857), and the research ethics committees of the national institute for medical research development (IR.NIMAD.REC.1400.102). COPSIR is a computer-based telephone interview survey operated by humans. In each survey, 515 adults were selected at random and interviewed by trained interviewers over three to four days. The surveys were conducted at approximately weekly intervals for the first four surveys, after which the intervals grew longer due to the spread of the pandemic-related changes. The details of the study protocol can be found elsewhere.^12^

###  Data Collection

**Figure 2:**
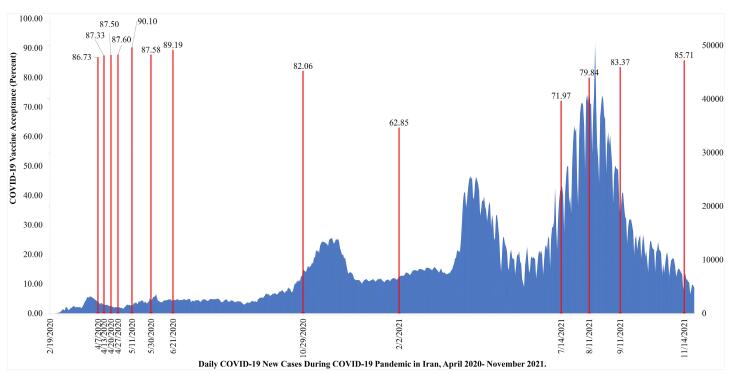
COVID-19 Daily New Cases and the COVID-19 Vaccine Acceptance Trend in Iranian Adults During 13 Waves of COPSIR Study, April 2020–November 2021

 The present study examined the COVID-19 vaccine acceptance trend and socio-demographic predictors across the 13 waves of COPSIR. Figure 2 illustrates the timeline of data collection surveys. The primary outcome of this study is based on responses to the statement, “If a vaccine that has become available is recommended for me, I will receive it”. This item assessed COVID-19 vaccine acceptance on a 7-point Likert scale ranging from 1 (strongly disagree) to 7 (strongly agree).

 In order to facilitate understanding of the vaccine acceptance trend, a cut-off was established to categorize the degree of acceptance as a binary variable. The scores 1 to 4 were interpreted as disagreement, while scores 5 to 7 were interpreted as agreement with the COVID-19 vaccination. All 13 surveys collected data on socio-demographic variables, including age, gender, education, marital status, residence (urban, rural), and also prior diagnosis of COVID-19. Moreover, the COVID-19 vaccine uptake was collected from the 10th wave, since the COVID-19 vaccine became available in Iran.

###  Data Analysis

 The chi-square test for trend was used to evaluate the trend of COVID-19 vaccine acceptance across the 13 surveys of COPSIR. We utilized a multilevel logistic regression with a random intercept to examine the effects of individual-level and survey wave variables on the COVID-19 vaccine acceptance rate. Multilevel logistic regression analysis was employed to account for the hierarchical structure of the data. Indeed, the individual units of analysis at a lower level are nested within the survey’s wave units at a higher level. Further, the COVID-19 vaccine acceptance in Iranian adults in each of the 13 waves of COPSIR was plotted based on demographic variables. In each survey, unadjusted and adjusted multilevel regression analyses were carried out to establish the odds of COVID-19 vaccine acceptance based on socio-demographic variables such as age, gender, education, marital status, residence, and prior diagnosis of COVID-19. In light of the previous contradictory research findings regarding the role of socio-demographic variables in the vaccine acceptance odds ratio (OR), we opted to include all variables in the adjusted model. Additionally, we examined the association between COVID-19 vaccine acceptance and COVID-19 vaccine uptake in each wave from 10 to 13 using the chi-square test. All analyses were conducted at a 5% level of significance using the Stata software version 14.1.

## Results

 This study investigated the COVID-19 vaccine acceptance rate in 13 consecutive surveys. The ﬁrst survey was conducted on April 7, 2020, and the last on November 14, 2021 (Figure 2). A total of 6,556 adults participated in the 13 surveys of COPSIR. Of them, 52.12% were male, 25.29% had an academic degree, 87.54% were married, 77.88% resided in urban areas, and 91.0% had history of confirmed COVID-19. The mean age of participants was 40.86 (Standard deviation [SD] = 12.27). There was no significant difference in the socio-demographic characteristics of the participants and prior diagnosis of COVID-19 during the 1st to 13th waves of the COPSIR study (Supplementary file 1).

 The results of chi-square for trend showed that the Iranian adults’ acceptance of the COVID-19 vaccine changed significantly over the course of the 13 surveys of COPSIR (4.47; *P* = 0.035). During the first eight surveys, the acceptance rate had a relatively steady trend: 86.73%, 87.33%, 87.50%, 87.60%, 90.10%, 87.58%, 89.19%, and 82.06%, respectively. In the ninth survey, conducted in February 2021, the rate dropped to 62.85%. Then, it rose gradually over the next four surveys, from 71.97% to 85.71% (Figure 2, Table 1).

**Table 1:** COVID-19 Vaccine Acceptance Trend in Iranian Adults During 13 Waves of the COPSIR Study, April 2020–November 2021

**Survey Number**	**COVID-19 Vaccine Acceptance** ** (percentage)**	**COVID-19 Vaccine Acceptance ** **Lower 95% CI**	**COVID-19 Vaccine Acceptance ** **Upper 95% CI**
1	86.73	83.81	89.66
2	87.33	84.42	90.24
3	87.50	84.60	90.40
4	87.60	84.70	90.50
5	90.10	87.50	92.71
6	87.60	84.67	90.48
7	89.19	86.49	91.90
8	82.06	78.64	85.49
9	62.85	58.59	67.11
10	71.97	68.03	75.91
11	79.84	76.33	83.35
12	83.37	80.13	86.61
13	85.71	82.67	88.76
Total	83.25	82.35	84.16

CI, confidence interval.

 Multilevel logistic regression analysis demonstrated a significant association between age and vaccine acceptance. Compared to the 18- to 25-year-old age group, the odds ratio of vaccine acceptance was 1.58 for the 46- to 55-year-old age group and 1.96 for the over 56-year-old age group. Additionally, acceptance of the COVID-19 vaccine was greater among those with a higher level of education. The vaccine acceptance odds ratios were 1.43 (CI:1.05‒1.96) and 1.60 (CI:1.16‒2.02) for individuals with academic degrees and high school and equivalent diplomas compared to illiterate individuals. Vaccine acceptance did not correlate with gender, marital status, residence, and history of COVID-19 (Table 2).

**Table 2:** Participant Characteristics and Results from the Unadjusted and Adjusted Multilevel Logistic Regression Analysis of Predictors of COVID-19 Vaccine Acceptability in Iranian Adults

**Variables**	**N (%)**	**COVID-19 Vaccine Acceptance Percent (SD)**	**Unadjusted OR**	**95% CI**	**Adjusted OR**	**95% CI**
Gender						
Female	3139 (47.9)	82.99 (0.38)	Ref.		Ref.	
Male	3417 (52.1)	83.49 (0.37)	1.05	(0.93‒1.21)	0.97	(0.85‒1.12)
Age group						
18-25	622 (9.5)	79.58 (0.40)	Ref.		Ref.	
26-35	1766 (26.9)	82.39 (0.38)	1.23	(0.97‒1.55)	1.16	(0.90‒1.49)
36-45	2075 (31.7)	82.17 (0.38)	1.24	(0.99‒1.56)	1.19	(0.92‒1.54)
46-55	1271 (19.4)	85.44 (0.35)	1.58	(1.22‒2.03)	1.58	(1.19‒2.10)
56 +	822 (12.5)	87.23 (0.33)	1.84	(1.38‒2.45)	1.96	(1.41‒2.71)
Education						
Illiterate	384 (5.9)	81.77 (0.39)	Ref.		Ref.	
Primary & Secondary	2252 (34.3)	83.57 (0.37)	1.100	(0.82‒1.47)	1.34	(0.99‒1.81)
High school & Diploma	2262 (34.5)	82.80 (0.38)	1.04	(0.78‒1.39)	1.43	(1.05‒1.96)
University	1658 (25.3)	83.78 (0.37)	1.15	(0.85‒1.54)	1.60	(1.16‒2.22)
Marital status						
Married	5739 (87.5)	83.62 (0.37)	Ref.		Ref.	
Unmarried	817 (12.5)	80.66 (0.40)	0.80	(0.66‒0.96)	0.87	(0.70‒1.08)
Area of residence						
Urban	5106 (77.9)	82.90 (0.38)	Ref.		Ref.	
Rural	1450 (22.1)	84.48 (0.36)	1.08	(0.92‒1.28)	1.14	(0.96‒1.35)
Prior diagnosis of COVID-19						
No	591 (9.0)	83.37 (0.37)	Ref.		Ref.	
Yes	5965 (91.0)	82.06 (0.38)	1.13	(0.89‒1.43)	1.11	(0.87‒1.40)

CI, confidence interval; Ref., Reference.

**Figure 3:**
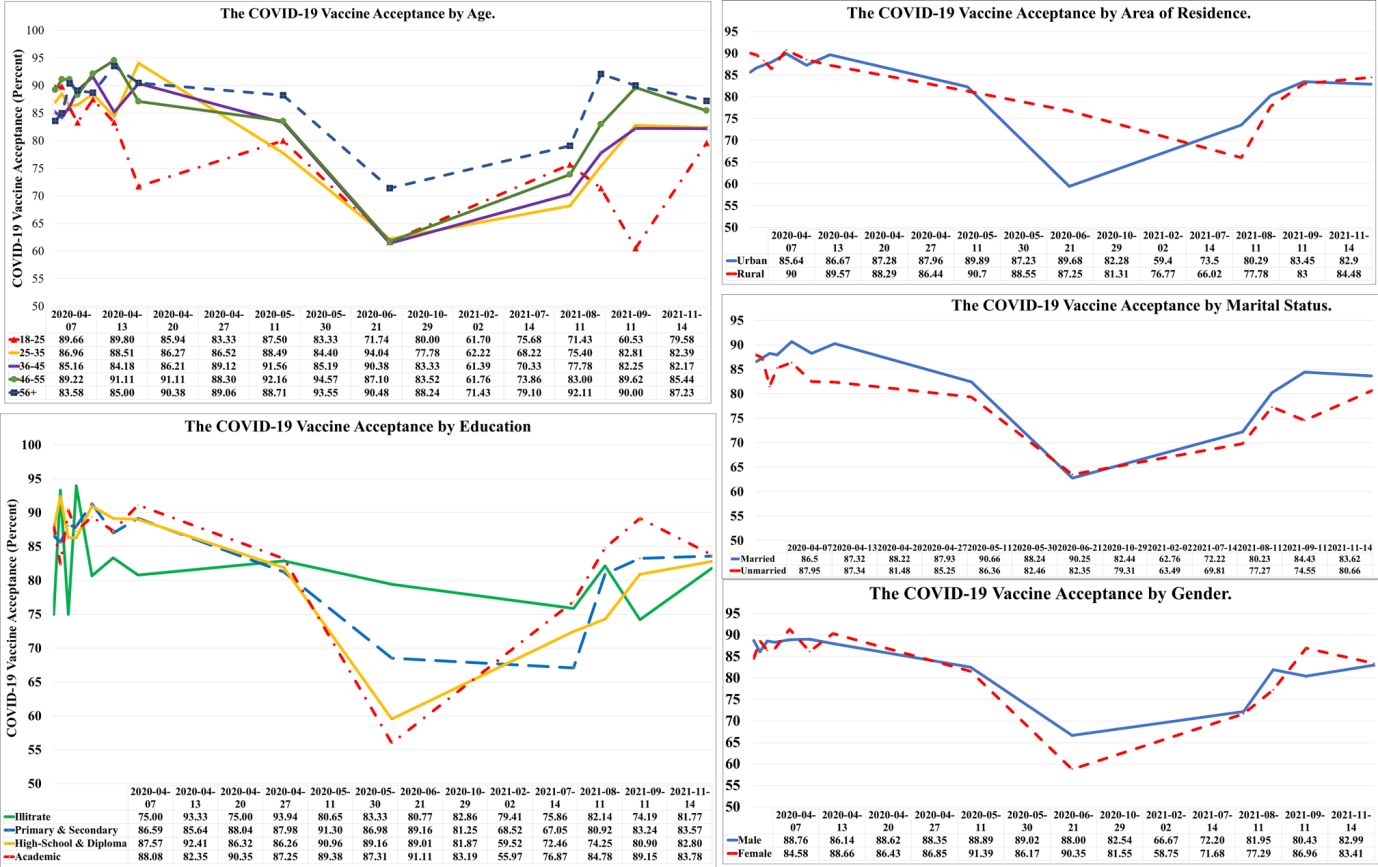
COVID-19 Vaccine Acceptance Trend in Iranian Adults During 13 Waves of COPSIR Study by Socio-demographic Variables, April 2020–November 2021

Figure 3 illustrates the COVID-19 vaccine acceptance trend among Iranian adults over the 13 waves of COPSIR as per socio-demographic variables. Unlike other surveys in this study, the 9th survey found a significant difference in the subgroups of residence. Acceptance of the COVID-19 vaccine was significantly lower in urban areas than rural areas (59.40% versus 76.77%; OR = 0.44; CI: 0.25‒0.75). In contrast to our overall finding, the acceptance of the COVID-19 vaccine and education were inversely related in the ninth survey. Higher education was associated with a lower COVID-19 vaccine acceptance; therefore, the odds of vaccine acceptance in academic and high school and equivalent education levels were (OR = 0.33; CI: 0.13‒0.81) and (OR = 0.38; CI: 0.16‒0.90), respectively, compared to illiteracy. There seems to have been a qualitative interaction between education and survey time. The decline in vaccine acceptance in wave 9 was not significantly different across age and gender subgroups.

 The results of the chi-square tests showed that in all waves from 10 to 13, there was a significant association between COVID-19 vaccine acceptance and uptake; this means that people who are more willing to vaccinate against COVID-19 actually vaccinate more (Table 3).

**Table 3:** Relationship Between COVID-19 Vaccine Acceptance and COVID-19 Vaccine Uptake in the 10th to 13th Waves of the COPSIR Study

**Wave**	**COVID-19 Vaccine Acceptance**	**COVID-19 Vaccine Uptake**	***P***** Value**
10	No	0 (0.0)	< 0.001
	Yes	31 (8.6)	
11	No	3 (2.9)	< 0.001
	Yes	146 (36.1)	
12	No	6 (7.1)	< 0.001
	Yes	211 (49.5)	
13	No	44 (60.3)	< 0.001
	Yes	412 (94.1)	

## Discussion

 It is now acknowledged globally that the only way to end the COVID-19 pandemic is to mass-produce and distribute its vaccines. Our research revealed that COVID-19 vaccine acceptance was relatively stable over the first eight surveys, with an acceptance rate of over 80%. Vaccine acceptance dropped sharply to 62.85% in February 2021 (9th survey) due to a period effect but gradually increased to 85.71% over the next four surveys. During the COVID-19 pandemic, approximately 20% of Iranian adults were reluctant to receive the vaccine. Vaccine hesitancy is a global phenomenon, and evidence indicates that global vaccine acceptance fell from 70% in March 2020 to less than 50% in October 2020.^6,13^ A survey conducted in June 2020 in 19 countries with 13 426 participants revealed that China, Brazil, and South Africa had the highest COVID-19 acceptance rates with 88.62%, 85.36%, and 81.58%, respectively, while Russia, Poland, and France had the lowest rates with 54.85%, 56.31%, and 58.89%, respectively. In June, vaccine acceptance was 71% in Kuwait and 65% in Canada, both of which utilized the COSMO questionnaire.^14,15^ At the same time, the acceptance rate was 87.58% in Iran. A study of adults’ attitudes toward vaccine acceptance in Arab countries estimated that the COVID-19 vaccine acceptance rate was, on average, 29.4% in December 2020. Among Arab countries, Kuwait, Jordan, and Saudi Arabia had the lowest acceptance rates with 23.6%, 28.4%, and 31.8%, respectively.^16^

 The COVID-19 vaccine acceptance was at its lowest level in survey 9 (February 2, 2021) (62.85%). The sharp decline in COVID-19 vaccine acceptance among Iranian adults appears to have coincided with the widespread publication of news surrounding the safety of the Pfizer, BioNTech, Moderna, and AstraZeneca vaccines in Iran. The news sparked debates among Iranian officials, some physicians, and the media.^17-19^ The impact of published news on vaccine safety appears irrefutable.

 On February 17, 2021, approximately two weeks after the ninth survey, general vaccination began in Iran based on age prioritization. Figure 1 illustrates the national trend of daily and total doses of the COVID-19 vaccine. From February to mid-July 2021, there appears to have been a shortage of COVID-19 vaccines, which may have contributed to a rise in vaccine acceptance, beginning with survey number 10. This trend can be explained by Timothy C. Brock’s “Commodity Theory” developed in 1968.^20^ The theory explains consumer behavior when a product or service is scarce, asserting that a product’s value is proportional to its availability. In general, a scarce product is perceived to have a higher value than one that is readily available.

 Some previous studies have found that older age, female gender, being married, residing in urban areas, and a higher level of education are determinants of vaccination rates.^6,14,21,22^ Conversely, some studies have not reported a significant correlation.^10,23,24^ Our research demonstrated that vaccine acceptance was not associated with marital status or gender. In contrast, our findings revealed that vaccine acceptance is strongly correlated with older age and higher level of education. We found no significant link between residence and COVID-19 vaccine acceptance. In the ninth survey, conducted in February 2021, urban areas demonstrated a greater decline in vaccine acceptance. We also identified a qualitative interaction between survey timespan and level of education in terms of effects on COVID-19 vaccine acceptance. Changes in vaccine acceptance among educated and urban residents in the ninth wave of the survey may be explained by their increased exposure to the COVID-19 pandemic and vaccine-related news.

 The theory of planned behavior (TPB), which is a consolidated conceptual framework, states that behavioral intentions are the most proximal determinants of health behaviors.^25^ This theory has recently been used on the willingness and uptake of the COVID-19 vaccine.^26^ It shows that the COVID-19 vaccine intention is influenced by various components and is itself a direct component in the COVID-19 vaccine uptake. During waves 10 to 13, we found a significant link between COVID-19 vaccine acceptance and uptake, which was consistent with TPB.

 The relatively long interval between the 8th and 10th surveys is one of the study’s limitations. Perhaps more data could have been collected if the surveys had been conducted at shorter intervals. This study also possessed a number of merits. First, the current study is one of the few to have examined the long-term trend of vaccine acceptance among the Iranian adult population. Second, the study examined the factors influencing the acceptance of the COVID-19 vaccine in the Iranian adult population using a large representative sample size.

 In conclusion,we investigated the COVID-19 vaccine acceptance trend and its socio-demographic predictors in a large sample of Iranian adults over an extended period. Our findings revealed that COVID-19 vaccine acceptance remained above 80% except in February 2021, when the “period effect” reduced the rate to 62%. This period effect followed disturbing news regarding the safety of COVID-19 vaccines and a heated debate among officials, some physicians, and the media. In general, acceptance of the COVID-19 vaccine was significantly associated with older age and higher education but not with gender, marital status, or residence. Our findings highlight that government and health system actions for the proper management of the COVID-19 pandemic must be based on timely and accurate information about the community’s acceptance of the vaccine.

## Supplementary Files

Supplementary file 1. Comparison of the demographic characteristics of the participants in the 1st to 13th waves of the COPSIR study.
