# Supplementary material for: Monitoring the COVID-19 Vaccine Acceptance Trend and its Determinants Among Iranian Adults
Source: Arch Iran Med. 2023 Aug 1;26(8):427–33. doi: 10.34172/aim.2023.65 (PMC10685739; doi:10.34172/aim.2023.65)
Supplement: Supplementary file 1 — Comparison of the demographic characteristics of the participants in the 1st to 13th waves of the COPSIR study. [file aim-26-427-s001.pdf]

Arch Iran Med. August 2023;26(8):427-S1.
10.34172/aim.2023.65

Supplementary 1. Comparison of the demographic characteristics of the participants in the 1st to 13th waves of the COPSIR study.

Variables	1	2	3	4	5	6	7	8	9	10	11	12	13	P. Value*
**Gender**														**0.303**
Female	253 (48.7)	238 (47.1)	258 (51.2)	244 (48.3)	253 (50.7)	259 (50.9)	233 (48.0)	233 (48.0)	240 (48.2)	226 (44.9)	229 (45.3)	230 (45.0)	227 (44.4)	
Male	267 (51.4)	267 (52.9)	246 (48.8)	251 (50.2)	261 (51.7)	246 (49.3)	250 (49.1)	252 (52.0)	258 (51.8)	277 (55.1)	277 (54.7)	281 (55.0)	284 (55.6)	
**Age Group**														**0.254**
18-25	58 (11.2)	49 (9.7)	64 (12.7)	54 (10.8)	48 (9.5)	42 (8.4)	46 (9.0)	55 (11.3)	47 (9.4)	37 (7.4)	42 (8.3)	38 (7.4)	42 (8.2)	
26-35	138 (26.5)	148 (29.3)	153 (30.4)	141 (28.2)	139 (27.5)	141 (28.3)	151 (29.7)	126 (26.0)	135 (27.1)	129 (25.7)	126 (24.9)	128 (25.1)	111 (21.7)	
36-45	155 (29.8)	158 (31.3)	145 (28.8)	147 (29.4)	154 (30.5)	162 (32.5)	156 (30.7)	162 (33.4)	158 (31.7)	182 (36.2)	162 (32.0)	169 (33.1)	165 (32.3)	
46-55	102 (19.6)	90 (17.8)	90 (17.9)	94 (18.8)	102 (20.2)	92 (18.4)	93 (18.3)	91 (18.8)	102 (20.5)	88 (17.5)	100 (19.8)	106 (20.7)	121 (23.7)	
56+	67 (12.9)	60 (11.9)	52 (10.3)	64 (12.8)	62 (12.3)	62 (12.4)	63 (12.4)	51 (10.5)	56 (11.2)	67 (13.3)	76 (15.0)	70 (13.7)	72 (14.1)	
**Education**														**0.531**
Illiterate	28 (5.4)	30 (5.9)	16 (3.2)	33 (6.6)	31 (6.14)	30 (6.01)	26 (5.11)	35 (7.22)	34 (6.83)	29 (5.77)	28 (5.53)	31 (6.07)	33 (6.46)	
Primary & Secondary	164 (31.5)	181 (35.8)	184 (36.5)	183 (36.6)	184 (36.4)	169 (33.9)	166 (32.6)	160 (33.0)	162 (32.5)	173 (34.4)	173 (34.2)	173 (33.9)	180 (35.2)	
High School & Diploma	177 (34.0)	158 (31.3)	190 (37.7)	182 (36.4)	177 (35.0)	166 (33.3)	182 (35.8)	171 (35.3)	168 (33.8)	167 (33.2)	167 (33.0)	178 (34.8)	179 (35.0)	
University	151 (29.0)	136 (26.9)	114 (22.6)	102 (20.4)	113 (22.4)	134 (26.9)	135 (26.5)	119 (24.5)	134 (26.9)	134 (26.6)	138 (27.3)	129 (25.2)	119 (23.3)	
**Marital Status**														**0.115**
Married	437 (84.0)	426 (84.4)	450 (89.3)	439 (87.8)	439 (86.9)	442 (88.6)	441 (86.6)	427 (88.0)	435 (87.4)	450 (89.5)	440 (87.0)	456 (89.2)	457 (89.4)	
Unmarried	83 (16.0)	79 (15.6)	54 (10.7)	61 (12.2)	66 (13.1)	57 (11.4)	68 (13.4)	58 (12.0)	63 (12.7)	53 (10.5)	66 (13.0)	55 (10.8)	54 (10.6)	
**Area of Residence**														**0.051**

Urban	390	390	393	382	376	368	407	378	399	400	416	411	396
	(75.0)	(77.2)	(78.0)	(76.4)	(74.5)	(73.8)	(80.0)	(77.9)	(80.1)	(79.5)	(82.2)	(80.4)	(77.5)
Rural	130	115	111	118	129	131	102	107	99	103	90	100	115
	(25.0)	(22.8)	(22.0)	(23.6)	(25.5)	(26.3)	(20.0)	(22.1)	(19.9)	(20.5)	(17.8)	(19.6)	(22.5)

*Based on chi-square test
